# A 100,000 Scale Factor Radar Range

**DOI:** 10.1038/s41598-017-18131-1

**Published:** 2017-12-19

**Authors:** Pierre-Alexandre Blanche, Mark Neifeld, Nasser Peyghambarian

**Affiliations:** College of Optical Sciences, University of Arizona, 1630 E University Blvd., Tucson, AZ 85721 USA

## Abstract

The radar cross section of an object is an important electromagnetic property that is often measured in anechoic chambers. However, for very large and complex structures such as ships or sea and land clutters, this common approach is not practical. The use of computer simulations is also not viable since it would take many years of computational time to model and predict the radar characteristics of such large objects. We have now devised a new scaling technique to overcome these difficulties, and make accurate measurements of the radar cross section of large items. In this article we demonstrate that by reducing the scale of the model by a factor 100,000, and using near infrared wavelength, the radar cross section can be determined in a tabletop setup. The accuracy of the method is compared to simulations, and an example of measurement is provided on a 1 mm highly detailed model of a ship. The advantages of this scaling approach is its versatility, and the possibility to perform fast, convenient, and inexpensive measurements.

## Introduction

Electromagnetic (EM) properties such as radar cross section (RCS) or antenna gain are usually measured in anechoic chambers^1^. Even though the size of these chambers can be very spacious, there are situations where the object of interest is so large that it exceeds the dimensions of existing facilities. Computer simulation is a very helpful tool, but the processing time and memory requirement increase as the cube of the model size in regard to the wavelength for FDTD (Finite-Difference Time-Domain), making the solution intractable for large systems (>100λ linear size)^2^. Also, these complex codes, including method of moments (MoM), Physical Optics (PO), or Uniform Theory of Diffraction (UTD) can easily diverge or present artifacts that must be identified by other means^3,4^. Considering that Maxwell’s equations of wave propagation are invariant under dilatation transformation^5^, it is possible to make the measurement on reduced size models by using a proportionally shorter wavelength than the one employed in radar (radio detection and ranging). By conserving the scale factor and material properties between model and wavelength, the solution of the EM wave propagation is identical. Matching the EM properties of the original component requires the substitution of the materials with similar permittivity and permeability at the scaled wavelength. The advantage of the scaling approach is that it is easier to make the measurement on a smaller model than on the original object. In our case we demonstrate that our technique is faster (from weeks to hours), less expansive by 2 to 3 orders of magnitude, can be done on a tabletop, and its validity has been confirmed by computational simulation.

For over 70 years, engineers have used scale models of large and/or complex radio-frequency (RF) systems to dertermine their EM properties. However, these models were limited to reduction factors of only 10–100 to remain in the same portion of the RF spectrum^6^. Using such a limited scale factor, provides the benefit that material properties are usually very similar for life ans scaled frequencies, and no substitution is required. However, the fabrication of large scale models is laborious, takes several weeks, and costs tens of thousands of dollars for a single model^7^. More recently, RCS measurement with a scale factor of a few hundreds and using terahertz frequency were demonstrated^8,9^. The advantage of this scale factor is that model can be fabricated by a variety of techniques such as CNC (Computer Numerical Control) mill, or additive 3D printing. Unfortunately, THz sources and detectors are not commonly accessible, and material properties in this frequency range have not yet been thoroughly measured^10^.

By carefully analyzing the advantages and shortcomings of different frequency domains to implement a compact range for RCS measurements, we selected the near infrared region (NIR), covering 700 nm to 2500 nm, as being the most promising in term of sources, detectors, fabrication techniques, as well as material properties. This spectral region benefits from the recent developments in photonics, plasmonic, and nano fabrication technology.

In this range of wavelengths, there exists a large variety of laser sources such as femtosecond pulsed fiber lasers that can be used for ranging^11^, or supercontinuum lasers that can be used for spectral analysis^12^. For wavelengths below 1.1 *μ*m, silicon based focal plane array detectors such as CCD and CMOS are readily available with high sensitivity, high resolution, and large pixel count. EM properties of materials are not only very well defined in the NIR, but organic chemistry and nanoparticles dopant can be used to obtain synthetic materials with good transparency and tunable refractive index^13^. These materials can be utilized to reproduce the permittivity observed at RF, such as rock and concrete (${\varepsilon ^{\prime} }_{r}=$ 2.5–5) using e.g. polymer doped with TiO_2_ nanoparticles^14^, or vegetation and foliage (${\varepsilon ^{\prime} }_{r}=$ 1.01–1.5) using e.g. silica aerogel with different pore sizes^15^.

With a scale factor on the order of 100,000 when moving from the center of the s-band (3 GHz) to 1 micron (300 THz), the accurate manufacturing of the model can be achieved by a variety of techniques. Among them, multiphoton additive manufacturing is extremly versatile and can have a resolution on the order of 100 nm, which correspond to full-scale feature sizes on the order of 10 mm^16,17^. Lithography and ion beam etching can also be used to achieve even better resolution (nm), although these techniques alone are much slower to fabricate the full model. Of course, other radio wavebands of interest including C, X. Ku, K, and Ka can be simulated by scaling the size of the model by the relevant ratio.

A different frequency range that was potentially interesting was thermal IR, or long wavelength IR (LWIR). Centered on 10 micron, LWIR has a scale factor of 10,000 which relaxes the tolerance on the model fabrication. Another advantage of operating in the LWIR is the availability of laser sources and high density thermal bolometer detectors^18^. Unfortunately, transparent dielectric materials are not common for that wavelength region. Only some of the chalcogenide crystals such as germanium have the suitable EM characteristics. Moreover, crystals are not compatible with the 3D printing process, which make them unsuitable for model fabrication. A summary of the advantages and limitations of the different EM regions are presented in Table 1.

**Table 1:** Advantage (+) and limitation (−) of the different EM regions for radar range.

Frequency scale	Fabrication techniques	Source	Detector	Material
GHz 1–100	(−) Too large, slow and expensive for structures of interest	(+) Good RF sources available	(−) Limited 2D FPA detector	(+) Very good material match
THz 100–1,000	(+) Many techniques available	(−) THz gap for sources	(−) THz gap for detectors	(−) Properties not well studied
LWIR 10,000	(+) Micron resolution	(+) Fiber and CO2 lasers	(+) Excellent (but expansive) thermo-bolometer detectors	(−) No suitable low loss dielectric
NIR 100,000	(−) Sub-micron resolution demanding but possible	(+) Large number of sources	(+) Ubiquitous CCD/CMOS Si detector <1:1 micron	(+) Polymer for low loss dielectric

## Experiment

**Figure 1:**
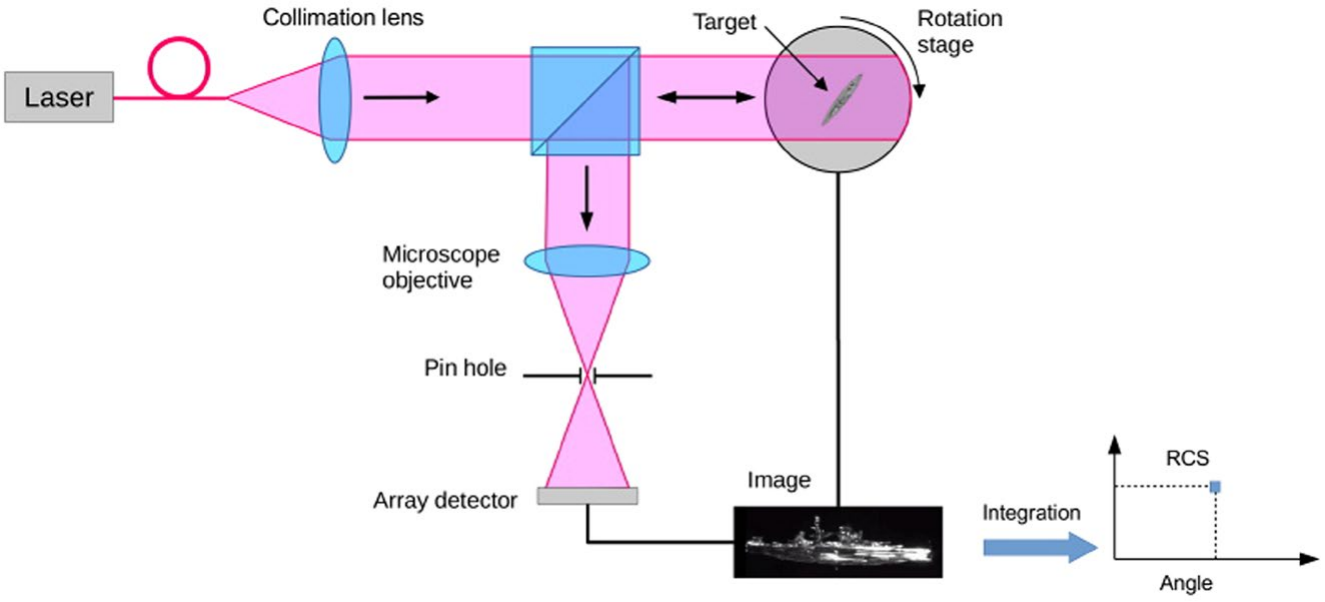
Monostatic RCS measurement setup. The target located on a rotation stage is illuminated with a CW 1064 nm laser. The back scattered signal is imaged on a CCD detector. The RCS value for a specific angle is obtain by integrating the intensity value of all the pixels in the image captured at that angle.

The experimental setup used to measure the RCS is presented in Fig. 1, and is based on a monostatic configuration where the target is illuminated along the same direction as the observed signal. The laser source is a CW 1064 nm single-frequency, narrow-linewidth (<5 kHz) fiber laser module, with 100 mW of output power, linearly polarized with an extinction ratio larger than 20 dB. At the output of the SMF fiber, a fiber collimator module is forming a beam with a 3.4 mm diameter (1/*e* ^2^). The light is directed toward the target by a 50/50 non-polarizing beam splitter located in front of the collection optics. The target is positioned on an automated rotation stage and can be aligned with respect to the beam and collecting optics with a 5-axes stage (x, y, z, θ, and φ). The back scattered signal is collected by a long working distance microscope objective, with either 100 × or 4 × magnification depending on the size of the target.

In a radar configuration a single RF detector would be located at the focal plane of the collection “optics”. In our case, instead of using a single cell photo-detector, we used an array detector placed at the image plane of the microscope objective. To reject the off-axis light that would have not be collected by a radar single cell photodetector, a 2 mm pin-hole was introduced at the focal plane of the microscope objective. This configuration is similar to a confocal microscope. The value of the RCS at a specific angle is obtained by integrating the intensity of all the pixels in the image captured at that angle.

Using a 2D detector instead of a single photocell has several advantages: first, the image can be used to determine the location of the scatterers responsible of the RCS signal (as shown in Fig. 1, before integration). Second, since the signal is distributed over the surface of the array detector, the overall dynamic range is multiplied by the number of pixels included in the image field. There are similarities between the information obtained using this setup and the inverse synthetic aperture radar (ISAR) technique: both provide 2D imaging data, yet our method does not require back projection computation.

## Results

To validate the experimental setup, we compared the results computed by an EM propagation software (ANSYS) to our measurement. For the software to give an accurate solution, we selected a shape simple enough so there would be no ambiguity in the simulation results. We also chose the size of the targets so there will be distinctive scattering features present in the RCS. This is obtained by operating within the resonant (Mie) scattering region, usually defined as 1 to 10 times the wavelength.

**Figure 2:**
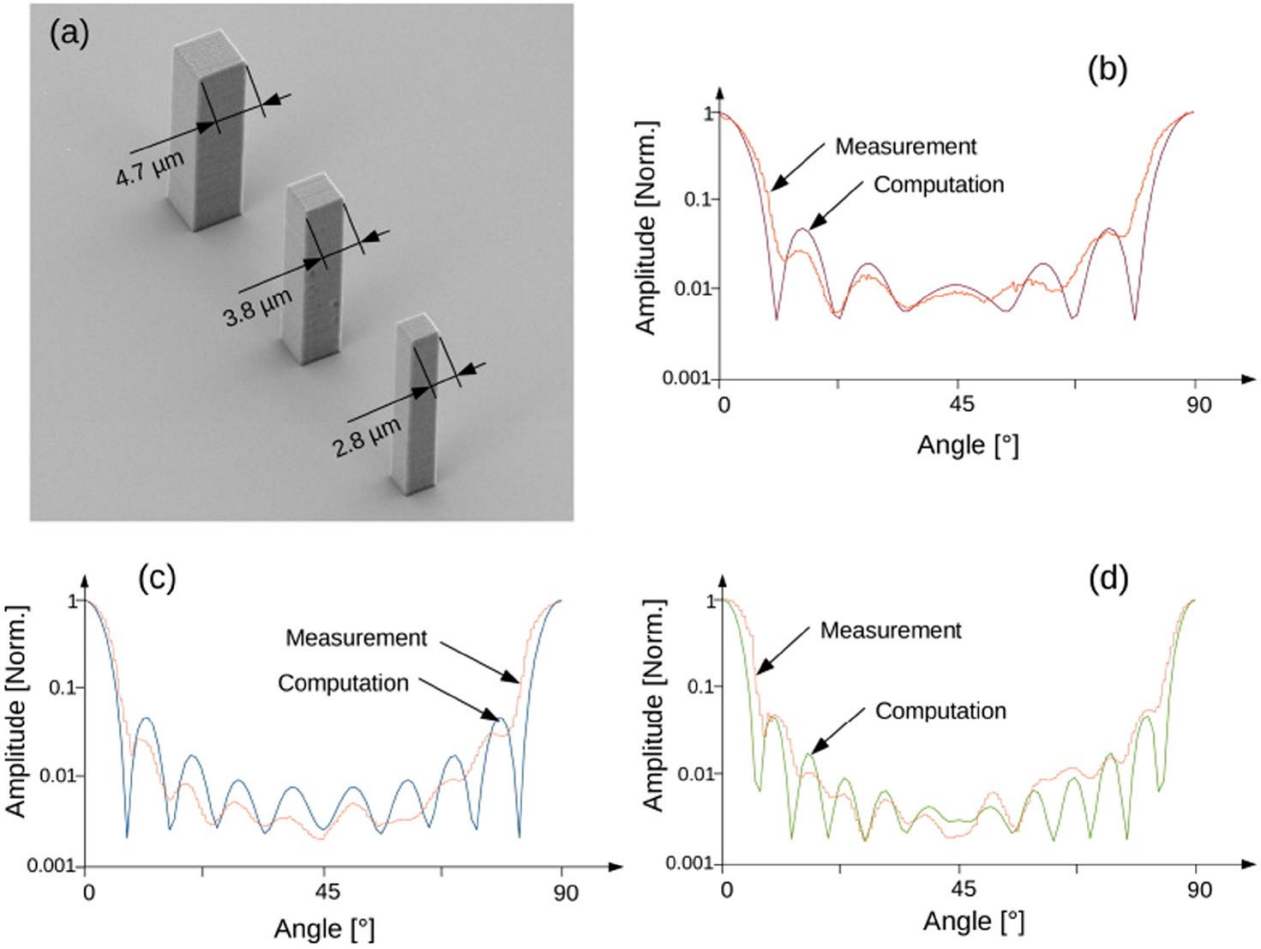
Validation of the experimental setup using micropillars of different sections. (**a**) SEM image of the micropillars fabricated by 3D printing. (**b**,**d**) Comparison between computation and measurement of the RCS for 2.8 *μm*, 3.8 *μm* and 4.7 *μm* section pillars respectively.

For this experiment, the targets are micro-pillars, 30 micron tall, with a square section of 2.8 micron, 3.8 micron and 4.7 micron. The samples were manufactured with a Photonic Professional GT 3D printer from Nanoscribe GmbH, using the highest possible resolution (160 nm). The section of the pillars has been measured with an electron microscope as presented in Fig. 2(a). The pillars were coated with 50 nm of gold to improve the reflectivity at 1 *μ*m wavelength.

The simulations were performed using ANSYS HFSS. The CAD model assumed perfect rectangular pillars (not reproducing the manufacturing artifact), and the material was perfect electrical conductor (PEC). We verified that the simulation gave identical results regardless of the frequency, providing that the scale factor between structure and frequency was respected, i.e: 300 *THz* with 3 micon size pillar, or 3 *GHz* with 0.3 meter size pillar.

Figure 2(b–d) present the comparison between simulation and experiment. Due to the geometry of the pillars, the RCS has a mirror symmetry every 45°. The amplitude of the measurement has been normalized and scaled to fit the computation. One can see that the measurements adequately reproduce the period of the oscillations present in the computation for all three samples. This agreement gives confidence that the technique is working adequately, even though subject to manufacturing artifacts or material mismatch such as metal conductivity.

**Figure 3:**
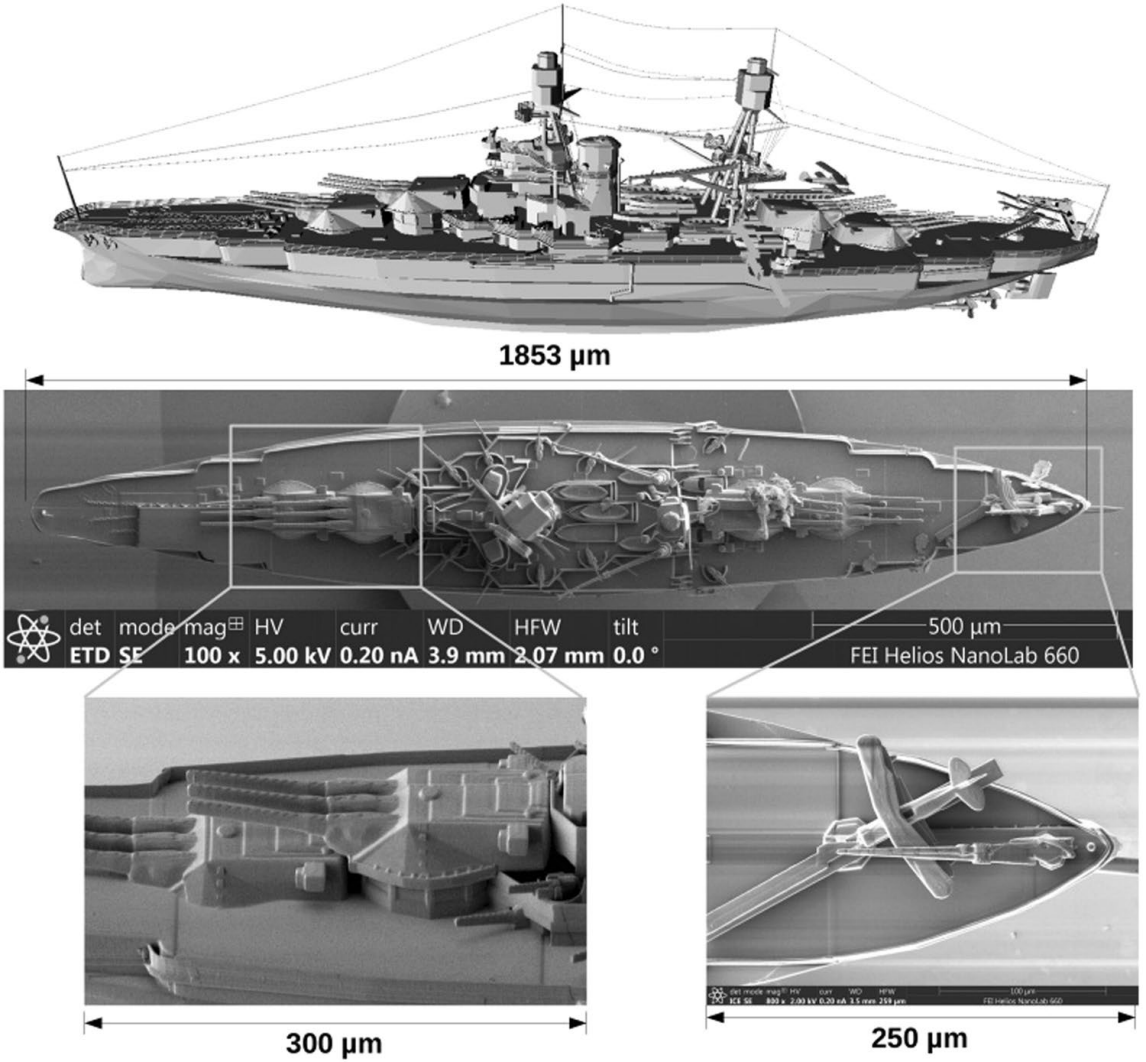
Top: Model of the USS Arizona. Middle: SEM image of the 3D printed model with 100,000 scale. Bottom: SEM images showing detail and resolution of the 3D printed model.

To demonstrate that the technique can be applied to more complex objects, we measured the RCS of the battle ship USS Arizona (BB-39). The 3D CAD file of this ship is available in the public domain^19^, and was used to fabricated a 100,000 scaled replica by 3D printing. The entire model required only 3 hours to print with the Nanoscribe Professional GT printer, and cost ≈ 100 USD in machine operation charges. SEM images of the reproduction are presented in Fig. 3 and show the details of the high resolution model. The entire structure was coated with gold to reproduce the high reflectivity of steel in the radio frequency domain at near IR.

**Figure 4:**
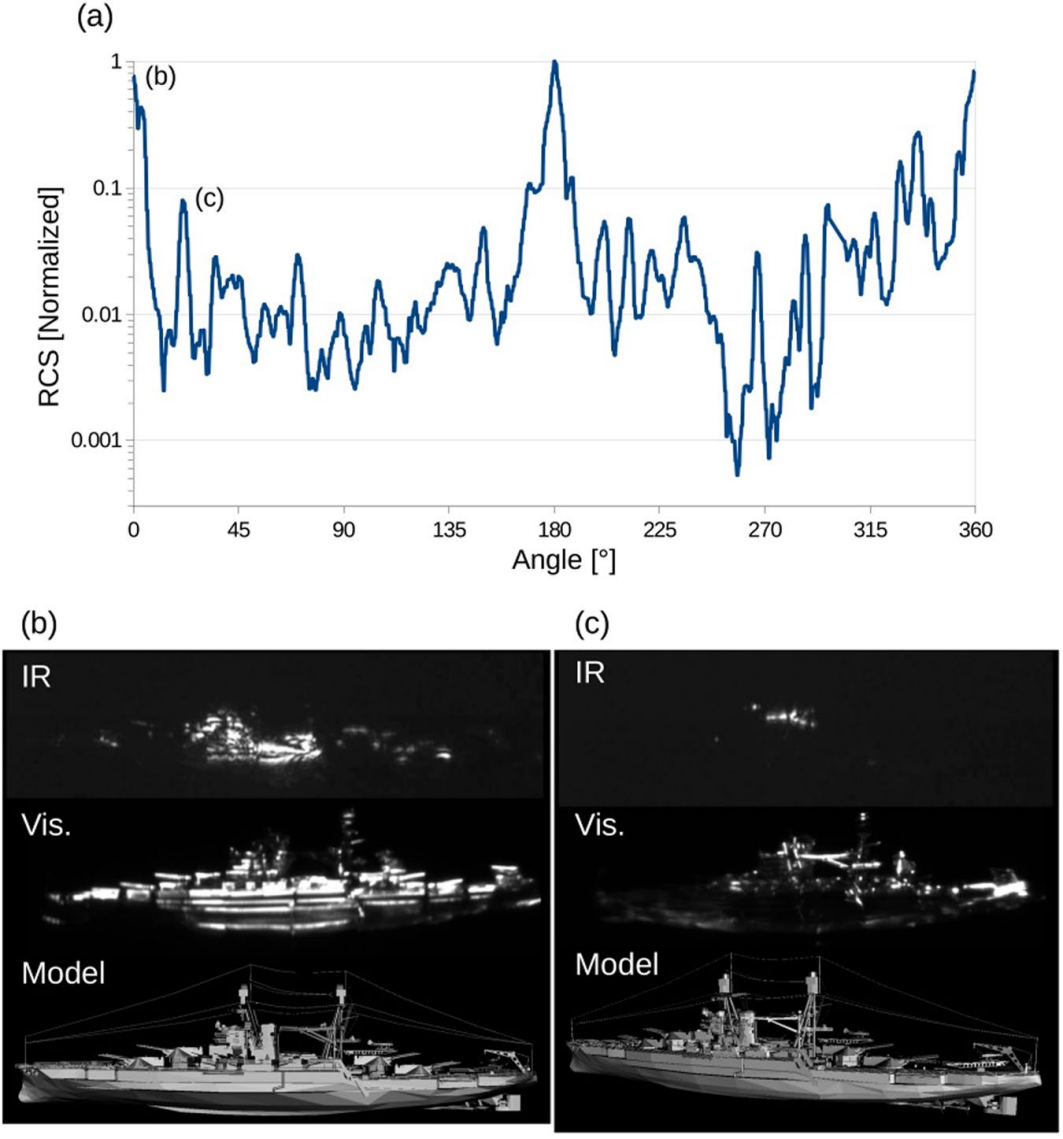
RCS of the 1:100,000 USS Arizona model measured at 1064 nm. (**a**) measurement obtained by integration over the image pixels. (**b**) and **(c**) images obtained at two different angles (top: NIR coherent source, middle: visible incoherent source, bottom: CGI model) for the identification of the scatterer responsible of the respective peaks.

The measured RCS of the USS Arizona model is presented in Fig. 4(a). Usually the identification of the dominant scatterers responsible for the RCS signal is not an easy task. However, in our approach, it becomes an obvious exercise since the image associated with a specific angle can be retrieved for inspection. Two examples are presented in Fig. 4(b,c), where we also compare the original images obtained in the IR with the coherent source, to images taken with a visible extended incoherent source (incandescent light bulb), and using the CAD model. Figure 4(b) is associated with large peak presents at 0° which is due to the reflection from the hull of the ship. Figure 4(c) is associated with the moderate peak presents at 21° which is due to a crane located mid-ship, port-side.

It was not possible to compared the experimental results obtained with the USS Arizona model with computation, because of the size of the model with respect to the wavelength was much too large ($1.2\times 1E\mathrm{6\ }{\lambda }^{2}$). This ratio required a unreasonable amount of computer RAM to be processed (estimated to be at least 150 Tbytes).

## Sea clutter

Another area of interest where a compact model radar range can provide significant advantage is the measurement of the RCS under noisy environments such as sea clutter^20^. Sea clutter is especially difficult to reproduce with an RF model range due to the size of the ocean patch needed to be statistically significant. In our case, with a scale factor of 100,000, the required sample only measures 5×5 mm and can easily be fabricated using the same 3D printing technique as mentioned before.

**Figure 5:**
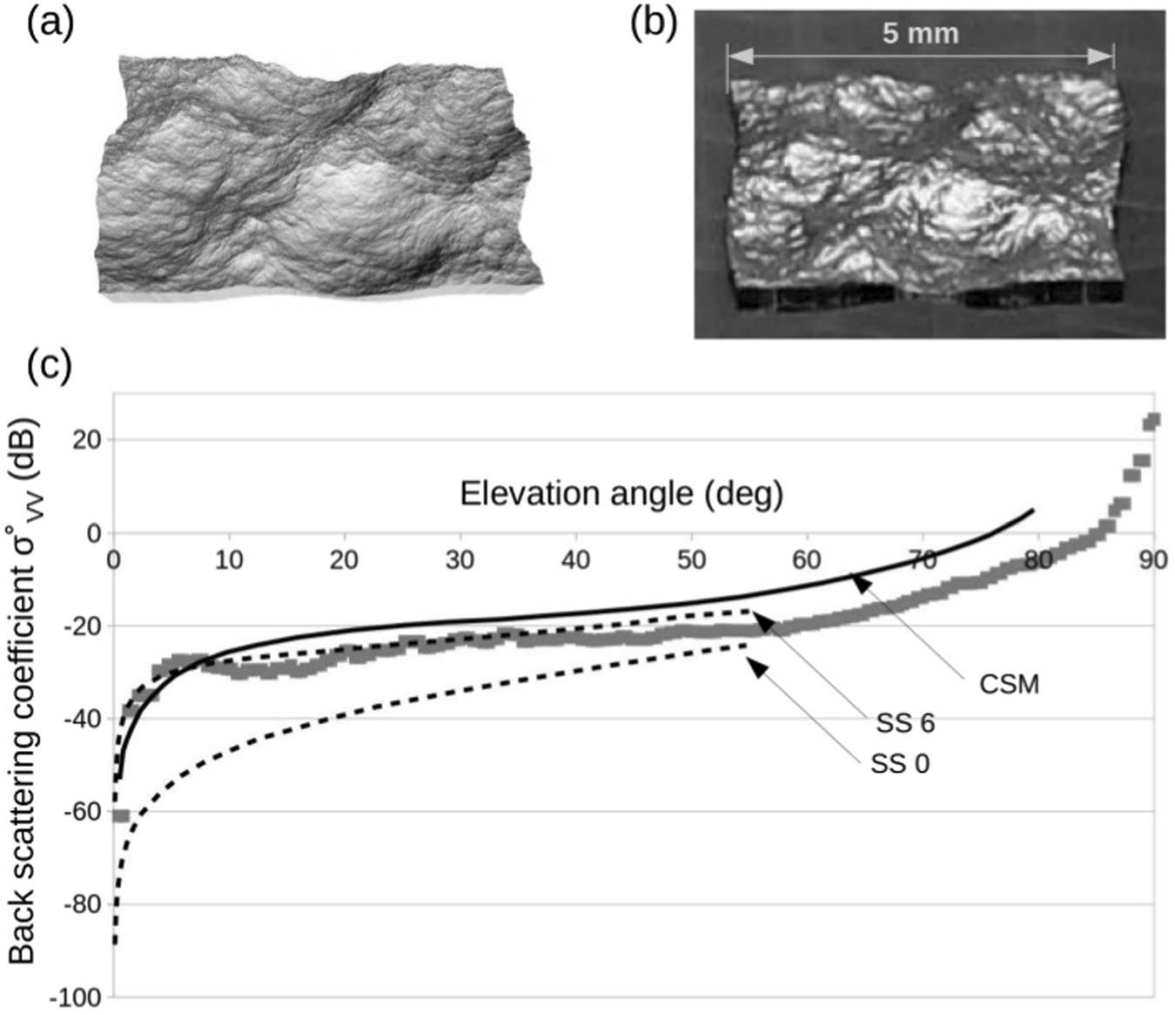
Sea clutter. (**a**) CGI model of the sea. (**b**) SEM image of the 3D printed sea patch. (**c**) Back scattering coefficient according to the altitude angle for vertical polarization. Lines are values obtained with two different NRL sea clutter models: doted lines are from ref.^25^ for sea state 0 and 6 at 3 GHz. Plain line is from ref.^24^ at 4 GHz. Data points are measurements from this work.

The see clutter model we used was generated using the ocean modifier in the open 3D creation software Blender^21^. This ocean modifier is a port from the open source Houdini Ocean Toolkit and has options for a variety of parameters such as wind speed and direction^22^. An image of the model we generated is presented in Fig. 5(a). An picture of the manufactured ocean patch is shown in Fig. 5(b) for comparison. The sample was coated with 50 nm of gold to reproduce the sea water reflection in the s-band.

We measured the backscattered coefficient *σ*° with our setup using vertical polarization (${\sigma }_{VV}^{0}$). The calibration was done using normal reflection from a gold coated flat plate which RCS is theoretically defined as:

1 $$\sigma =\frac{4\pi {A}^{2}}{{\lambda }^{2}}$$

where *A* is the area of the plate, and *λ* the observation wavelength.

At 3 GHz, a 1m^2^ plate has an RCS (*σ*) and backscattered coefficient (*σ*°) of 36 dB. By comparison to this value, the measurement we obtained with the ocean model give a backscattered coefficient of 24 dB which is consistent with real life measurement^23^.

In Fig. 5(c), we compared our measurements to two NRL sea clutter models. The plain line is the prediction from the composite surface model (CSM) at 4 GHz^24^, when the doted lines are from ref.^25^ generated at 3 GHz for sea state 0 and 6. A good agreement between the models and the measurement was obtained.

## Discussion and Conclusion

In this article, we demonstrated a compact RCS range with a scale factor of 100,000. The range uses 1 *μ*m wavelength and leverages nanoscale resolution 3D printing to timely and accurately manufacture the models. The back scattered signal is collected on a FPA detector which provides 2D imaging capability that can be used to identify the scatterers responsible for the RCS peaks.

The 3D printer we used in our experiment has a 3D lateral feature size specification of: “≤200 *nm*; typically 160 *nm*”. Considering the scale factor of 100,000 in our setup, this correspond to a size of ≤20 *nm* at the central s-band radar wavelength of 3 GHz. The printer also produces some mesa artifacts in the vertical direction that can be seen in the close view e-beam microscopy images presented in Fig. 3. This resolution limitation and artifacts certainly impact the accuracy of the RCS measurement. Although it is difficult to address the RCS accuracy in a general manner since it depends on the precise geometry of the model, it can be seen from the measurement on the micro pillars (Fig. 2) that the imperfections of the model, and the experimental setup, are washing out some of the feature of the RCS. Similarly, we expect that some details of the RCS obtained with the more complex model were smoothed out. It is possible to imagine that other techniques can be used in addition to nano 3D printing to improve the model resolution. Example of such techniques are lithography and focused ion beam. We are actively pursuing this direction to add important EM elements to our model such as emitting antennae.

It is also expected that the substitution of the conductive material either steel or sea water for gold could affect the measurement due to different skin depth effect. During the simulations with the micro-pillars, we tested this hypothesis by varying the conductivity (*σ*) of the material from perfect electrical conductor (PEC: $\sigma =\infty \,S/m$) to different metals (silver $\sigma =6.3\times 1E7\,S/m$, steel $\sigma =1E6\,S/m$), or even an imperfect metal with reduced conductivity of $\sigma =1E2\,S/m$. It was observed that a reduction of the conductivity by a factor 1E4 (steel to imperfect metal) reduced the amplitude of the RCS by less than 3% (at 3 GHz frequency). However, this reassuring number cannot be expected to apply for all possible model configurations. Some possible mitigation can be obtained by using different coatings on the scaled model such as silver or aluminum that have lower reflectivity than gold in the near infra-red. This strategy would need to be implemented on a case by case basis.

From a material perspective, it should also be noted that since we are using NIR wavelength, high transmission polymeric materials can be employed to reproduce the permittivity of most dielectric encountered at GHz frequency. This advantage can be critical to reproduce rock and concrete that are an important component for land and urban environment.

These advantages and limitations should be put into perspective to other techniques such as large model ranges, or computation. The large model ranges used by the Navy at SPAWAR has a scale factor of 1/50^*th*^, is using single material (brass), and is also impacted by manufacturing artifacts and limited accuracy. Considering this specific scale factor, the SPAWAR range should use models with an accuracy better than 0.5*mm* to achieve similar feature size.

Simulations also have their own limitations in resolution and are subject to computational artifacts. FDTD, which is among the most precise computational algorithm for electromagnetisme (EM), is prone to the well documented staircasing error^26^. Precise EM computational methods such as MoM, PO, or UTD are also known for their limitation to handle large and highly detailed models due to the increase in computational time and memory requirement.

In the future, the proposed approach can be extended to ranging measurements by using a short pulse laser source and a gated detector, or by exploiting time of flight interferometry. We also envision a setup where plasmonic nanoantennas are implanted directly into the model to measure antenna gain, shadowing, as well as interference^27,28^. Such an active system can be useful to optimize the antenna location in the case of the future 5 G wireless communication systems, for which signal accessibility is important^29,30^.
